# Editorial: The role of autophagy in infectious diseases

**DOI:** 10.3389/fcimb.2022.1039282

**Published:** 2022-09-23

**Authors:** Hua Niu, Meihong Deng

**Affiliations:** ^1^ Laboratory of Hepatobiliary and Pancreatic Surgery, Affiliated Hospital of Guilin Medical University, Guilin, China; ^2^ Guangxi Key Laboratory of Molecular Medicine in Liver Injury and Repair, Guilin Medical University, Guilin, China; ^3^ Guangxi Health Commission Key Laboratory of Basic Research in Sphingolipid Metabolism Related Diseases, Affiliated Hospital of Guilin Medical University, Guilin, China; ^4^ Center for Immunology and Inflammation, The Feinstein Institutes for Medical Research, Manhasset, NY, United States; ^5^ Department of Molecular Medicine, Zucker School of Medicine at Hofstra/Northwell, Manhasset, NY, United States

**Keywords:** autophagy, infectious diseases, pathogens, autophagic regulation, host defense

## Introduction

Autophagy is an intracellular catabolic process that sequesters and digests cytosolic components to maintain cellular homeostasis in health and diseases (Biasizzo and Kopitar-Jerala, 2020). Canonically, a series of evolutionarily conserved proteins identified as autophagy-related proteins participate in the autophagy process, forming double-membrane autophagosomes in the cytosol to digest the cytosolic components (Tsukada and Ohsumi, 1993). Autophagy has been reported to play critical roles in clearing intracellular pathogens and modulating the inflammatory response during host defense (Deretic et al., 2013). These make the autophagy process as an attractive target for developing therapeutic strategies for infections.

However, emerging studies indicate that the autophagy process is complicated and appears to be pathogen-specific. First, three forms of autophagy, macroautophagy, microautophagy and chaperone-mediated autophagy, are identified in mammalian cells according to diverse mechanisms of transporting cellular materials into lysosomes. Second, alternative mechanisms bypassing the canonical autophagy pathway have been recognized in the autophagy process in response to pathogen invasion. Furthermore, the autophagy process crosstalks with other cellular activities to modulate the host defense. Finally, some pathogens can hijack or exploit the autophagy process for their invasion. Therefore, further studies are urging to understand the pathogen-specific mechanisms of autophagic regulation during infection and the crosstalk between autophagy and other host defense mechanisms.

In this Research Topic issue, we have collected a series of research articles, reviews and perspective on recent advances in the mechanism of autophagic regulation in response to bacterial and viral infections, as well as autophagy in other aspects.

## Advances in autophagy during bacterial infection

Here we have collected two research articles, three reviews, and one perspective covering the role of canonical and noncanonical autophagy pathways in bacterial infection. The research article by Nikouee et al. showed that activating Beclin-1 in mice by forced expression of active mutant *Becn1F121A* or by treatment of Beclin-1–activating peptide enhanced autophagy and alleviated adverse outcomes of pneumonia-induced sepsis. This work shows the therapeutic potential in treating pneumonia-induced sepsis by activating Beclin-1. Pellegrini et al. reviewed studies on autophagy in host defense against an intracellular microorganism, *Mycobacterium tuberculosis*, in tuberculosis patients. Although Autophagy is known to mediate pathogen clearance and plays an important role in the control of inflammatory response during bacterial infections, some infectious agents, such as *Salmonella* Typhimurium, have developed mechanisms to escape or hijack autophagy for their benefit. One known mechanism is *via* direct interactions between the effector proteins secreted from the pathogen and autophagy proteins. Using a computational network analysis approach, Demeter et al. have identified and validated that the *Salmonella* Pathogenicity Island-1 effector protein, SpoE, directly interacts with the host SP1 transcription factor. Furthermore, SpoE negatively regulates the expression of the autophagy-related protein, MAP1LC3B and modulates the autophagy flux during *S.* Typhimurium infection in intestinal epithelial cells. Flores-Vega et al. summarized the principal strategies used by *Pseudomonas aeruginosa* and *Burkholderia cenocepacia* to escape or hijack microbicidal mechanisms within the autophagic pathway in cystic fibrosis patients. Besides the canonical autophagy pathways, non-canonical autophagy pathways, such as LC3-associated phagocytosis (LAP) and Pore-Forming Toxin-Induced Non-Canonical Autophagy (PINCA), are implicated in host defense during bacterial infection. Grijmans et al. reviewed the recent advance in LAP against bacterial pathogens. Herb et al. discussed the molecular differences and similarities between LAP, PINCA and xenophagy, a selective form of macroautophagy, in macrophages during bacterial infections.

## Advances in autophagy during viral infection

Autophagy is implicated in the life cycle and resistance of virus infection. Here Mauthe et al. showed that HSBP1, a very small cytoplasmic coiled-coil protein, interacts with FIP200-ATG13-containing complexes to control the stability of ULK complex for autophagy induction and picornaviral replications in U2OS cell lines. Li et al. showed that Interferon alpha 2a (IFNα-2a), a treatment for chronic Hepatitis B virus (HBV) infection, interplays with the Akt/mTOR signaling and AMPK signaling to regulate the autophagy process and HBV replication under various glucose concentrations. These findings may help improve the therapeutic efficacy of IFNα-2a in treating HBV infection. Matsui et al. reviewed and highlighted the interaction between Hepatitis C virus (HCV) NS5A protein and hepatocyte-nuclear factor 1α (HNF-1α) together with the chaperone protein HSC70 to promote the lysosomal degradation of HNF-1α *via* chaperone-mediated autophagy (CMA), resulting in HCV-induced pathogenesis. These call for further investigations of HCV NS5A-interacting proteins containing CMA-targeting motifs to understand HCV-induced pathogenesis. Severe acute respiratory syndrome coronavirus 2 (SARS-CoV-2) has caused the pandemic in the past three years without effective treatment. Here Silva et al. summarized the interplay between coronaviruses and autophagy regarding virus life cycle, cell resistance, and inflammation and discussed the autophagy-targeted pharmaceuticals being tested in clinical trials with distinct mechanisms. Autophagy is not exclusive to animals or humans. It also happens in other organisms. Wu et al. summarized the mechanisms of how virus evades host immune responses by disrupting and manipulating host autophagy in plant and animals. Picot et al. reported that autophagy is activated in hemolymph and the mantle of pacific oysters in response to infection by the virus OsHV-1. This study may help find solutions for mortality outbreaks of young Pacific oysters, which have seriously affected the oyster-farming economy in several countries worldwide.

## Advances in autophagy in other aspects

The research article by Zhou et al. investigated the roles of Atg1 and Atg13 homologs in a nematode-trapping filamentous fungus, *Arthrobotrys oligospora.* The authors characterized the phenotypes in Atg1 mutant and Atg13 mutant strains. They found that compared to the wild type, these two mutants both are defective in autophagosome formation, highlighting the crucial roles of these Atg genes in the autophagy process in *A. oligospora*. Furthermore, they showed Atg1 contributes to other phenotypes, such as sporulation and nematode predation, indicating the additional roles of Atg1 in the growth and development of *A. oligospora*.

Autophagy and nitroxidative stress both promote the clearance of invading pathogens and intertwine with each other. TLR4 activates autophagy and causes nitroxidative stress through downstream signal pathways after engaging pathogen-associated molecular patterns (PAMPs). The review article by Zhang et al. summarized signaling pathways that connected TLR4, autophagy, and nitroxidative stress in infectious diseases, and discussed their triangular relationships that affect cellular homeostasis.

## Conclusion and perspective

This Research Topic provides updated knowledge into current understanding about the interaction between host autophagy and pathogens. Further studies are required to understand the pathogen-specific autophagy pathways and the crosstalk between autophagy and other signaling pathways in the host against pathogen infections.

## Author contributions

All authors listed have made a substantial, direct, and intellectual contribution to the work and approved it for publication.

## Conflict of interest

The authors declare that the research was conducted in the absence of any commercial or financial relationships that could be construed as a potential conflict of interest

## Publisher’s Note

All claims expressed in this article are solely those of the authors and do not necessarily represent those of their affiliated organizations, or those of the publisher, the editors and the reviewers. Any product that may be evaluated in this article, or claim that may be made by its manufacturer, is not guaranteed or endorsed by the publisher.
